# Errata

**Published:** 1881-02

**Authors:** 


Errata.—Circumstances were not favorable for a careful revision of the proof of the last issue, and we were compelled to rely upon the compositor, or foreman in the publication office, for the performance of that important duty. While the errors are more numerous than usual they are chiefly typographical, and due to the omission of letters or their transposition by the printer, and do not effect the matter to any material extent. They are chiefly as follows, viz:

Page 1, *rl* is omitted in *particularly*. Page 2, *e*, instead of *i*, should be in *antedating*. Page 12, 7th line from bottom, read *wanting* for *water*. Page 14, read *lymphatic* for *sympathetic*. Page 15, Oct. 8, read *wild* for mild. Page 16, for 116½ read from 100 to 106½. On same page, second column, 2d line, read *accompanied* for accampanied. Page 19 read *exacerbation*. Page 20, many things, read *many twinges;* persevering should read *presuming*. Page 21, *calomel* for colomel. Page 24, Amorphous. Page 21, 25th line, read severely. Page 24, for asthma read *asthenia*. Page 25 read pustulent. Page 28 read circumvallation, ostitis, liquefaction. Page 29, preceded cotton absorbing; *no requisition is made*, may be erased altogether in second line from the bottom of the page. Page 31 Laertes; page 33 valueless; page 36 their stemming: page 37 inferred; page 40 medicine; page 43 formulæ. The final *e* in Dr. Osbourn's name should be omitted.

The foregoing are the errors for which we are compelled to crave the indulgence of our readers. We promise to be more careful in the future.

